# What evidence is there for a delay in diagnostic coding of RA in UK general practice records? An observational study of free text

**DOI:** 10.1136/bmjopen-2015-010393

**Published:** 2016-06-28

**Authors:** Elizabeth Ford, John Carroll, Helen Smith, Kevin Davies, Rob Koeling, Irene Petersen, Greta Rait, Jackie Cassell

**Affiliations:** 1 Division of Primary Care and Public Health, Brighton and Sussex Medical School, Falmer, Brighton, UK; 2 Department of Informatics, University of Sussex, Falmer, Brighton, UK; 3 Division of Medicine, Brighton and Sussex Medical School, Falmer, Brighton, UK; 4 Research Department of Primary Care and Population Health, UCL, London, UK; 5 Department of Clinical Epidemiology, Aarhus University, Denmark

**Keywords:** Rheumatoid arthritis, electronic health records, data quality, general practice, free text

## Abstract

**Objectives:**

Much research with electronic health records (EHRs) uses coded or structured data only; important information captured in the free text remains unused. One dimension of EHR data quality assessment is ‘currency’ or timeliness, that is, data are representative of the patient state at the time of measurement. We explored the use of free text in UK general practice patient records to evaluate delays in recording of rheumatoid arthritis (RA) diagnosis. We also aimed to locate and quantify disease and diagnostic information recorded only in text.

**Setting:**

UK general practice patient records from the Clinical Practice Research Datalink.

**Participants:**

294 individuals with incident diagnosis of RA between 2005 and 2008; 204 women and 85 men, median age 63 years.

**Primary and secondary outcome measures:**

Assessment of (1) quantity and timing of text entries for disease-modifying antirheumatic drugs (DMARDs) as a proxy for the RA disease code, and (2) quantity, location and timing of free text information relating to RA onset and diagnosis.

**Results:**

Inflammatory markers, pain and DMARDs were the most common categories of disease information in text prior to RA diagnostic code; 10–37% of patients had such information only in text. Read codes associated with RA-related text included correspondence, general consultation and arthritis codes. 64 patients (22%) had DMARD text entries >14 days prior to RA code; these patients had more and earlier referrals to rheumatology, tests, swelling, pain and DMARD prescriptions, suggestive of an earlier implicit diagnosis than was recorded by the diagnostic code.

**Conclusions:**

RA-related symptoms, tests, referrals and prescriptions were recorded in free text with 22% of patients showing strong evidence of delay in coding of diagnosis. Researchers using EHRs may need to mitigate for delayed codes by incorporating text into their case-ascertainment strategies. Natural language processing techniques have the capability to do this at scale.

**Strengths and limitations of this study**

- A study using general practice patient records from Clinical Practice Research Datalink, representative of the UK population.
- 218 000 words of medical free text were annotated in triplicate by domain experts, with adjudication by a senior clinician.
- We did not mark negation or modifiers within the text, so it is unknown what influence these linguistic features would have on our understanding of the results.
- Data were collected between 2005 and 2008 and it is not clear how changes to clinical practice in the last decade may have influenced recording.

## Introduction

Electronic health records (EHRs) are increasingly used for epidemiological research, clinical audit and service evaluation in chronic conditions, such as rheumatoid arthritis (RA).^1–4^ For example, UK general practice patient records from the Clinical Practice Research Datalink (CPRD) have been used to audit treatment of RA in the population,^5–7^ to evaluate the accuracy of rheumatoid factor test^8^ and to assess the risks to patients while using disease-modifying antirheumatic drugs (DMARDs) and biological therapeutic agents.^9^ ^10^

### General practice EHRs

In the UK, 98% of the population is registered with a general practitioner (GP), and GP operates as the gatekeeper to all secondary care health services. GPs record all facets of care the patient receives in electronic patient records, including correspondence to and from hospital specialists. GPs record using a medical coding system called Read codes,^11^ ^12^ a hierarchical system covering diagnoses, symptoms, tests, referrals, administrative codes and correspondence, as well as free text notes. GPs record prescriptions using British National Formulary or product codes.

### Data quality in general practice patient records

Data quality in EHR data is thought to comprise several dimensions such as completeness, correctness, concordance, plausibility and currency or timeliness.^13^ ^14^ Data are considered current or timely if they are recorded in the EHR within a reasonable period of time following measurement and they are representative of the patient state at the time of measurement. Currency has been assessed the least in validation studies of EHR data.^13^

The majority of studies using general practice patient record data use only coded information. If diagnostic coding is delayed, research using general practice patient records using only codes may miss cases, as codes are not ‘representative of the patient state at the time of measurement’.^15^ The issues of delayed diagnostic codes may primarily affect conditions where diagnoses are made in secondary care, such as RA, as the transfer of information can lead to slippage in primary care coding.

The validity of inflammatory arthritis codes in UK general practice electronic patient records is thought to be good; that is, if there is an arthritis code, it is highly likely that the patient has the disease.^16^ However, no validation studies of RA in UK general practice records have assessed the sensitivity of RA codes, or their timeliness in application.

### GPs' use of text in patient records

The way that GPs use Read codes varies, but many describe choosing a ‘summary’ code which is a keyword representing the main body of the consultation.^17^ The GP may then add text under the code to capture complexity, evolving circumstances, uncertainty and severity.^18^ Some examples of codes and the accompanying free text are shown verbatim in table 1. Letters received from specialists can also be added to the record as free text.

**Table 1:** Read codes from general practice patient records and examples of accompanying free text

Code	Accompanying text
N06z.11 Arthritis	Generally worse—quite immobile at times—knees and wrists swollen
N245.14 Hand pain	Pain in small joints in both hands—some hurt more than others. Wrists also in problem. O/E—tender over MCP and PIP joints of the index finger in both hands. Wrist movement painful. No joint swelling
6A…00 Patient reviewed	Increased joint pains, concerned if SE of hydroxychloroquine. Pain in shoulders and hands on mobilisation—more likely to be Rh A, awaiting appointment at rheumatology
8C1B.00 Nursing care blood sample taken	FBC, ESR, UE, SLFT, fasting glucose, TFT, CHOL, CRP, PSA, urate
1992.00 Vomiting	Since waking this morning. No haemoptysis. O/E—looks pale and unwell. Abdomen soft, bowel normal. Had a motion this morning/normal. Stop prednisolone and indometacin. Twice omeprazole and take gaviscon (QDS). Do Hb/ESR/CRP to check progress of arthritis. Symptoms of arthritis completely disappeared. No history of indigestion

CHOL, cholesterol; CRP, C reactive protein; ESR, erythrocyte sedimentation rate; FBC, full blood count; Hb, haemoglobin; MCP, metacarpophalangeal; O/E, on examination; PIP, proximal interphalangeal; PSA, prostate specific antigen; Rh A, rheumatoid arthritis; SE, side effect; SLFT, liver function test; TFT, thyroid function test; UE, urea and electrolytes.

### The use of free text to identify delays in coding

Previous studies using CPRD data to investigate delays in diagnosis of ovarian cancer in UK general practice found that 45% had text indicating a definite diagnosis of cancer, and in 22% this was before the coded date.^19^ ^20^ No such investigation has previously been conducted in RA, but there is evidence from US-based EHR studies that free text can make RA case ascertainment more sensitive. A study using US hospital records to find cases of RA found that a single International Classification of Diseases, ninth revision (ICD-9) disease code for RA had a positive predictive value (PPV) of only 19%, a combination of coded data achieved a PPV of 56% and an algorithm incorporating text attained a PPV of 94%.^21^ The addition of text to the algorithm increased the sample size by 26%.^21^

### Why should we investigate delay in coding RA?

RA affects between 0.5% and 1% of the population,^22^ ^23^ and is characterised by joint swelling, joint tenderness and destruction of synovial joints, leading to severe disability.^24^ RA is an autoimmune condition, and it is recognised that its early active management with treatment by DMARDs and biologic therapies can slow progression to disability.^25^ ^26^ The UK National Institute for Health and Care Excellence (NICE) issued guidance in 2009 for the treatment of RA, recommending early intervention with DMARD combination therapy,^27^ and CPRD studies have been used to evaluate the population-wide adherence to these guidelines.^5–7^ The care pathway for patients with RA in the UK varies by region, but frequently there is a referral from general practice, diagnosis and management recommendations by hospital specialists, which are then communicated back to general practice for ongoing management.^28^ In a previous study, we found that the free text keywords ‘rheumatoid arthritis’ were most commonly found associated with the Read codes ‘Letter from specialist’, ‘Seen in rheumatology clinic’ and ‘Incoming mail NOS’,^17^ suggesting that diagnostic information contained in letters was not coded at the time of receipt of the letter. We argue that if RA diagnoses are not coded in a timely way, UK general practice records will lack ‘currency’, and studies such as Edwards *et al*^5^ will not reflect accurately the treatment of RA in the UK.

We thus investigated delay in coding of RA in UK general practice patient records by analysing the free text, with two aims:

- To quantify RA-relevant information found in free text, in terms of its quantity, timing and association with Read codes.
- To estimate delayed diagnostic coding of RA, by using DMARD in text as a proxy for RA diagnosis. These medications are generally only initiated by rheumatologists following definitive diagnosis of RA, after which prescriptions are continued in primary care, and may therefore be a good diagnostic marker.

Our hypothesis was that DMARDs mentioned in text prior to diagnostic code would be a marker for an RA diagnosis that had not yet been coded.

## Methods

### Ethics statement

This research was approved by the UK MHRA Independent Scientific Advisory Committee, protocol no. 09_033R.

### Study design

This was an observational study of routinely collected UK general practice data.

### Data sources and study population

The CPRD is an electronic database of anonymised longitudinal patient records from general practice (http://www.cprd.com). Established in 1987, it is a UK-wide data set covering 8.5% of the population, with data from over 600 practices. It is broadly representative of the UK population, with 5.2 million currently active patients.^29^ Records are derived from the general practice computer system VISION (In Practice Systems; http://www.inps4.co.uk/) and contain complete prescribing and coded diagnostic and clinical information as well as information on tests requested, laboratory results and referrals made. Quality is assured by various assessments, including the practice-level ‘up-to-standard’ assessment which is derived from 10 data quality parameters.

### Read codes

Read codes are a hierarchically structured vocabulary developed by a UK GP, Dr James Read, in the 1980s. They map to other nomenclatures such as ICD codes and International Classification of Primary Care (ICPC) codes. Each Read code represents a term or short phrase describing a health-related concept. There are over 200 000 different codes, which are sorted into categories (diagnoses, processes of care and medication) and subchapters.^30^ Each clinical entity is represented by a 5-byte alphanumeric code and a Read term which is the plain language description.

### Identification of cases

From the target population of permanently registered patients in the study period of 1 January 2005–31 December 2008, 294 cases were identified at random who had a first diagnostic code of RA within the study period (code list published elsewhere^31^), were aged 30 years and over at the time of diagnosis and who had records available from ‘up-to-standard’ practices, from 1 year before the first coded diagnosis of RA to 14 days afterwards. This sample size was chosen as the maximum number feasible to obtain given limitations on acquiring and handling such a large volume of text (218 000 words), and given anonymised free text is only available from CPRD at considerable additional cost.

### Development of indicator code groups

We drew up lists of codes that were indicative of RA-related activity in the record (indicator code groups) based on clinical consultation and code list dictionaries. These were then modified by reviewing the codes actually used in the records of patients with RA before the diagnostic code was found in their records. This process (published elsewhere; ref. 31) generated eight indicator code groups of interest for the current study: *(1) disease-modifying antirheumatic drug* (*DMARD) prescription, (2) referral to rheumatology specialist, (3) initial inflammatory arthritis diagnosis*, *(4) rheumatoid factor test, (5) synovitis, (6) joint signs and symptoms, (7) other arthritis diagnosis and (8) non-specific inflammatory marker tests*.

### Free text data

All text strings in the records were accessed, from a period beginning with the first of 1 year before the first RA code, the first DMARD prescription or the first specific marker date (if earlier than 1 year); up to a maximum of 2 years before the first RA code. The period ended 14 days after the first RA diagnostic code. All text strings were manually anonymised in a three-stage process at CPRD before being released to the research team.

### Information extraction from text

Information was extracted from text by manual review. All text strings were triple annotated by senior medical students following standardised guidelines. Annotators used an annotation workbench created with the Visual Tagging Tool software^32^ which allowed relevant strings to be highlighted and assigned a category or subcategory. Disagreements between annotators were adjudicated by a senior clinician (JCas).

### Categorisation of free text strings

The free text was mapped onto the indicator code groups (table 2): *(1) rheumatoid arthritis; (2) diagnosis or sign of inflammatory arthritis, (3) rheumatoid factor test (subcategories: test done, positive result, negative result), (4) referral to rheumatology, (5) synovitis, (6) DMARD prescription; (7) other arthritis symptoms or diagnosis, (8) non-specific inflammatory marker tests and (9) joint pain or swelling symptoms (subcategories: pain, swelling/inflammation, movement/stiffness, effusion)*.

**Table 2:** Rheumatoid arthritis indicator categories for annotation

Main category	Examples
Rheumatoid arthritis	▸ Rheumatoid* (eg, rheumatoid lung, rheumatoid disease)
A diagnosis or sign of inflammatory arthritis	▸ Polyarthritis ▸ Polyarthropathy ▸ Palindromic rheumatism ▸ Rheumatic arthritis ▸ Psoriatic or gouty arthropathy ▸ Arthritis or arthropathy linked to infection
Rheumatoid factor test Test done NOS Positive result Negative result	▸ Latex test ▸ Heterophile ▸ IgA agglutination test ▸ Rose-Waaler test ▸ Rheumatoid factor level ▸ RAHA test
Referral to rheumatology	▸ Rheumatology disorder monitoring ▸ Rheumatology treatment change ▸ Rheumatology management plan given ▸ Under care of rheumatologist
Synovitis	–
DMARD prescription	▸ Separate table given for drug names
Other arthritis symptoms or diagnosis	▸ Allergic arthritis ▸ Transient arthropathy ▸ Unspecified polyarthropathy ▸ Unspecified monoarthritis ▸ Other specified arthropathy ▸ Arthritis NOS
Immune/inflammatory markers that are not specific to RA	▸ Antinuclear factor ▸ Antimitochondrial autoantibody ▸ Antismooth muscle autoantibody ▸ Parietal cell autoantibodies ▸ Autoimmune profile ▸ Antiliver kidney microsomal antibody level ▸ Plasma C reactive protein ▸ Serum C reactive protein level
Joint pain or swelling symptoms Pain Swelling/inflammation Movement/stiffness Effusion	▸ Knee pain ▸ Joint abnormal ▸ Joint swelling ▸ Reduced joint movement ▸ Joint movement painful ▸ Joint stiffness ▸ Inflammation in the joint ▸ Movement limitation ▸ Joint pain ▸ Joint effusion

*Indicates a 'wildcard' character where any term beginning with the keyword stem would be accepted for the search.

Arthritis of the MCP is a distinguishing feature of rheumatoid arthritis.

DMARD, disease-modifying antirheumatic drug; IgA, immunoglobulin A; RA, rheumatoid arthritis; MCP joint, metacarpophalangeal joints.

### Statistical analysis

The number and timing of free text strings were tabulated. Non-parametric tests (Kruskal-Wallis) were used for comparison of median differences as data were found to be skewed. The final data were prepared and analysed using Stata V.11.2 (StataCorp LP, Texas, USA).

## Results

### Study population

In total, 6387 newly diagnosed cases of RA were identified between 2005 and 2008 comprising 2007 men and 4380 women. Of these cases, a random sample was extracted of 85 men of median age 65 years (IQR 55–75 years) and 209 women of median age 63 years (IQR 50–74 years).

### Relevant free text strings

In total, these 294 patients had 34 738 events recorded during the study period, of which 11 965 had text associated with the events (34.4%). Of these, strings were marked up with 4340 incidences of information related to RA in the 15 disease indicator categories prespecified (table 2).

### Quantity and timing of free text information prior to RA diagnosis

Table 3 shows the percentages of patients with disease information in text, codes and the proportion for whom information was only found in text. It also gives the median time intervals between the first text entry of each category in the patient record and the index date of RA diagnostic code (a positive value indicates event was *before* RA code). The earliest recorded information, on average, was rheumatoid (Rh) factor test with a negative result, other arthritis diagnoses and various joint signs and symptoms.

**Table 3:** Number and timing of free text string events compared with timing of coded information

Category	Patients with text entry (N) (%)	Patients with equivalent code (N) (%)	Patients with text only (N) (%)	Median time earliest text entry prior to RA code (days) (IQR)
DMARD	114 (39%)	131 (45%)	27 (9%)	38 (0–281)
Rheumatoid arthritis	121 (41%)	294 (100%)	–	34 (0–168)
Inflammatory arthritis	63 (21%)	33 (11%)	53 (18%)	140 (28–256)
Other arthritis	124 (42%)	41 (14%)	109 (37%)	148 (37.5–361)
Synovitis	54 (18%)	11 (4%)	48 (16%)	82 (16–206)
Referral to rheumatology	126 (43%)	122 (42%)	61 (21%)	68.5 (14–181)
Joint pain	193 (66%)	–	–	169 (52–356)
Effusion	28 (10%)	–	–	102 (15.5–278)
Stiffness	108 (37%)	–	–	116.5 (18.5–263)
Swelling	137 (47%)	–	–	148 (35–302)
Any joint symptom	221 (75%)	133 (45%)	106 (36%)	
Rh test, no result	94 (32%)	–	–	47.5 (17–154)
Positive Rh test	62 (21%)	–	–	66 (8–169)
Negative Rh test	25 (8.5%)	–	–	178 (11–334)
Any Rh test reference	135 (46%)	163 (55%)	26 (9%)	
Steroids	86 (29%)	–	–	119 (18–372)
Non-specific inflammatory markers	185 (63%)	181 (62%)	52 (18%)	120 (23–298)

DMARD, disease-modifying antirheumatic drug; RA, rheumatoid arthritis; Rh, rheumatoid factor.

### Association of text and codes

Table 4 shows the top 20 codes under which RA-relevant text information was found, representing 56.6% of all RA-related text information. In total, 24.7% of relevant text information was found under the top three codes, ‘letter from specialist’, ‘patient reviewed’ and ‘seen in rheumatology clinic’. The majority of information was found under communication codes (7 of 20 codes) and general consultation codes (3 of 20).

**Table 4:** Top 20 codes for all relevant text entries (total=4340)

Code	Frequency	% of all strings
Letter from specialist	486	11.20
Patient reviewed	296	6.82
Seen in rheumatology clinic	290	6.69
Pain in joint—arthralgia	228	5.26
Incoming mail NOS	191	4.40
Rheumatoid arthritis	182	4.20
Telephone encounter	119	2.74
Had a chat with patient	91	2.10
Erythrocyte sedimentation rate	70	1.61
Nursing care blood sample taken	56	1.29
Blood withdrawal	53	1.22
Arthritis	50	1.15
Pain	47	1.08
Seen by rheumatologist	47	1.08
Seen in hospital outpatients	44	1.01
Incoming mail	43	0.99
Hand pain	42	0.97
Knee pain	41	0.95
Communication from:	40	0.92
Foot pain	38	0.88

### DMARD in text as a proxy for RA diagnosis

#### How many patients have DMARD>14 days prior to their code?

**Figure 1:**
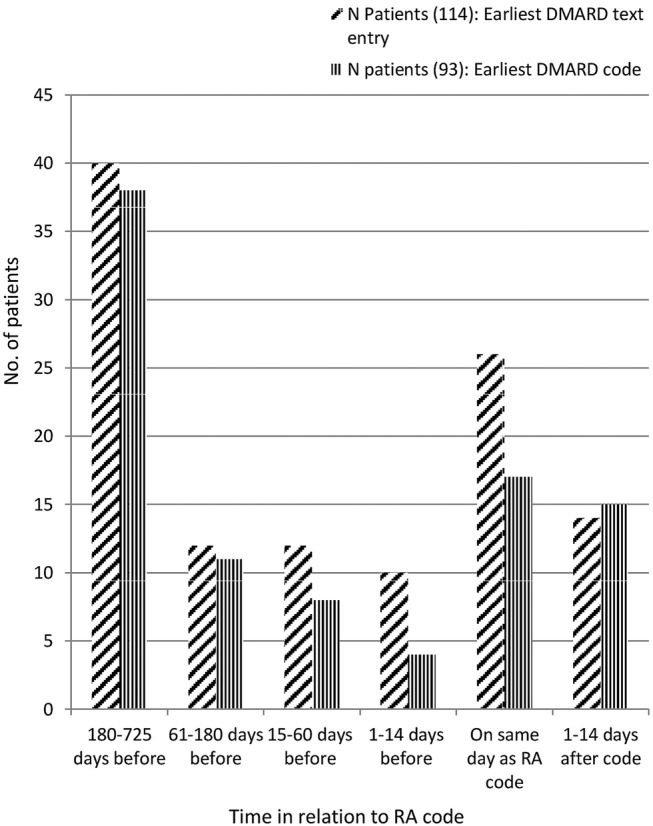
Timing of earliest DMARD text string or code. DMARD, disease-modifying antirheumatic drug; RA, rheumatoid arthritis.

The earliest date that DMARDs were found in each patient's record is shown in figure 1. Sixty-four patients (22%) had DMARD text entries earlier than 14 days before the RA code, and these patients formed the ‘early DMARD group’. Those without an early DMARD entry formed the ‘comparison group’. Early DMARD patients had a median of four text entries about DMARDS (max 68), spanning up to 725 days (2 years) prior to RA code.

#### Comparison with early DMARD codes

Fifty-seven patients had codes for DMARD more than 14 days earlier than the RA diagnostic code (19%). The intervals between earliest DMARD in text and RA code, and earliest DMARD code and RA code, were highly correlated (r=0.63, p<0.001), but the two groups of patients were not identical; 44 patients had early DMARDs in text and code (this was 77% of the group with early codes and 69% of the group with early text).

#### Evidence of delayed diagnostic recording in patients with early DMARD

The early DMARD group (N=64) did not differ from the comparison group in age or gender. They had more free text entries prior to their diagnostic code, a median of 20.5 vs 8 (Kruskal-Wallis test, p<0.001). In particular, they had more text entries prior to RA code referring to DMARDs, inflammatory arthritis, other arthritis, referrals to rheumatology and the symptom of swelling, than the comparison group (all p<0.002).

Text entries for disease indicators also occurred much earlier in the early DMARD group: inflammatory arthritis was mentioned first at a median of 192 days in the early DMARD group versus 78 days in the comparison group (p=0.03); for other arthritis diagnoses this difference was 281 vs 77 days (p=0.0001); for referral to rheumatology the difference was 218 vs 33 days (p=0.0001) and for swelling it was 245 vs 85 days (p=0.0001). RA was found in text at a median of 214.5 days prior to RA code in the early DMARD group compared with 14 days prior to RA code in the comparison group (p=0.0001); a positive Rh factor test was mentioned in text at a median of 186.5 days prior to RA code in the record vs 31 days for comparison group (p=0.007); and pain was also mentioned early, at a median of 251.5 vs 138 days (p=0.002).

Differences were not found in the *number of codes* that patients had, except that the early DMARD group had more DMARD prescriptions in their record. They had no greater number of codes for inflammatory arthritis or other arthritis diagnoses than the comparison group (p>0.05), although codes for these were reported earlier in the early DMARD group (inflammatory 147 vs 63.5 days, p=0.05; other arthritis 157.5 vs 56, p=0.048). Codes for referral to rheumatology (245 vs 16 days, p=0.0001) and Rh factor test (180 vs 35 days, p=0.0001) were found earlier in the early DMARD group.

## Discussion

We investigated the utility of free text for evaluating the EHR data quality dimension of ‘currency’. The results of our study suggest that free text information relevant to RA diagnosis was widespread and was found under correspondence codes, general consultation codes and general symptom codes. When information from text was added to the information in codes, 10–37% more patients were found to have evidence of each of the RA disease indicators that we investigated.

We showed good evidence for a group of patients who were likely to have a working diagnosis of RA, and were being treated with DMARDs, up to 2 years before the RA diagnosis was coded in primary care. These patients had significantly more information in text suggesting they had been through a formal diagnostic process (eg, referral to rheumatology, tests and RA keywords). These findings have implications for EHR research and for the delivery and planning of clinical care.

### Implications for EHR research

Very little previous work has sought to use information from text to establish delayed recording of diagnosis in EHR research. Tate *et al*^19^ ^20^ looked at free text for confirmation of a diagnosis of ovarian cancer, and showed that 22% of patients had text suggesting a diagnosis before the ovarian cancer code, a similar proportion to this study. In 10% of patients, this was over 4 weeks before the diagnosis date. The process of ovarian cancer diagnosis is similar to RA as the GP will refer the patient to specialists for diagnosis on suspicion of the condition, and the diagnosis will come back to the GP in a letter.

Free text has, however, been established as valuable in increasing the sensitivity of case-ascertainment strategies in EHR research^33^ and particularly in RA.^21^ Free text is already being employed in many studies using US hospital-based electronic records, and in some UK research groups, but extraction of free text information from UK general practice patient records is rare. Privacy and governance issues can be of concern, as free text is harder to strip of patient identifiers than structured data; however, the science of automated deidentification of text is now well established.^34^ Once text is anonymised, natural language processing algorithms can extract information from medical text at scale.

It could be speculated that within CPRD data, where there are delayed codes, there may also be codes that are completely missing. The results of this study do not give us any information about how many patients might never receive a diagnostic code for their RA, and furthermore, very few validation studies of CPRD data have assessed rates of false negatives or the sensitivity of case-ascertainment strategies. The Thomas *et al*^16^ study showed that codes for RA had high validity or PPV, but no attempt was made to assess sensitivity. Many EHR studies have explicitly ‘increased the probability of including only well-defined and pure cases’,^35^ that is, maximising specificity over sensitivity, which may be preferable in studies examining relative risks. However, this strategy may not be appropriate for estimating incidence, prevalence or healthcare audit studies where finding every case of disease is imperative. Developing and implementing methods for assessing sensitivity of case ascertainment in general practice record databases would lead to substantial improvements in our understanding of data quality and it is likely that use of free text will be a key part of such methods.

### Implications for clinical care

Patients with RA need active management for their condition, and getting the right care early in the disease process is critical. In general practice, this means prescribing the appropriate treatment for RA, assessing relevant comorbidities and their treatment, and taking care of the patient's general health and well-being. With the increase in shared care between different clinicians within the general practice, having diagnoses accurately coded is important. Since 2013, GPs in the UK have been financially incentivised to keep a register of their patients with RA to ensure care is given according to guidelines as part of the Quality and Outcomes Framework (QOF).^36^ The findings in this study suggest that during the study period (2005–2008), letters from specialists containing diagnostic information were either sometimes delayed in reaching the GP, or content of letters was not properly coded when received. However, since data collection in 2005–2008, there have been changes in service delivery and organisation, a change from paper based to electronic transfer of information between primary and secondary care, and the introduction of NICE and QOF guidance for RA. It is likely that coding quality has improved in recent years, so that examples of low-quality coding are over-represented in our sample and do not reflect current practice. Free text fields may not add as much information in 2016 as they did in 2008, but further study of more recent data is needed to explore this.

Another possible explanation for the apparent delay in coding is that there may be a genuine delay in establishing a definitive diagnosis, because RA is a heterogeneous disease. DMARDs may be initiated for a working diagnosis of inflammatory arthritis or other related inflammatory conditions. However, we found no evidence for more diagnoses of these inflammatory conditions in the records of the patients with probable delay, although there was more information in text regarding inflammatory arthritis. This adds further to the case that for conditions where diagnosis is a process that unfolds over time, much diagnostic information is found in the free text rather than the codes.

### Strengths and limitations of the study

A strength of this study was that the annotation was performed to high standard, in triplicate by domain experts, with adjudication by a senior clinician. This study used a sample of 294 patients, and produced a 218 000 word gold standard text corpus. This corpus could be used in the development of natural language processing algorithms which may be generalisable to other studies of RA in UK general practice patient record data.

A further strength is the data source, CPRD, which is believed to be representative of the UK population. However, despite random sampling, the small sample may not represent all patients with RA, or indeed all coding practices in the UK. The data also cannot inform us about other countries' primary care systems and recording of RA. Other European countries such as the Netherlands also extract and use data from primary care providers for research, but these systems use ICPC codes rather than Read codes. An investigation of delay in recording of cases of disease in these databases by using free text might also be profitable.

One further limitation is that negation and modifiers were not marked within the text, and the influence of these linguistic features on the results is unknown. Future work should assess the impact of modifiers and negation. However, if their impact is minimal, then keyword searches, which are quick and easy to specify, may be adequate to extract appropriate information from text.

## Conclusions

Electronic medical records have a key purpose in patient management and care, and are also now a key resource for research. Their primary purpose remains as a clinical record, but the secondary purpose of research can be successfully achieved with careful understanding of how diagnoses are recorded. This study shows that RA is coded as a diagnosis at variable intervals from the first indication of the disease. Late diagnostic coding has implications for the provision of routine care, especially for those with comorbidities and with ongoing high-risk medications. Text fields are an integral part of EHRs and researchers using EHR data should adapt their practice to take account of this additional and clinically important information, for example, by incorporating text into case-ascertainment strategies. Natural language processing techniques have the potential to extract information from text at scale.
